# The role of gender equity in healthy life expectancy in 27 European countries

**DOI:** 10.1093/eurpub/ckac129.163

**Published:** 2022-10-25

**Authors:** T Leão

**Affiliations:** EPI Unit, Instituto de Saúde Pública da Universidade do Porto, Porto, Portugal

## Abstract

**Background:**

The number of years - and healthy years - a person is expected to live differs according to gender: women tend to live longer than men, but with a shorter healthy life expectancy (HLE). Power imbalance, such as income, prestige, or autonomy gaps, may lead to an unequal health distribution across gender in the older ages. The association between gender equality and longer life expectancies has been described, but little is known about its association with HLE. We aimed to study the association between gender equality, and its components, and HLE in Europe, in the last decade.

**Methods:**

We combined HLE estimates from Eurostat with the Gender Equality Index (and its components) for 27 European countries, from 2013 to 2019. The associations between gender equality and HLE, and its gender gap, were assessed using regression analyses adjusted for Gross Domestic Product, number of medical doctors per 1000 inhabitants (as proxies of economic wellbeing and access to healthcare), and year.

**Results:**

Higher gender equality was associated with longer HLE in men (β = 0.22 p < 0.01), but not in women. Yet, higher equality in education was associated with longer HLE in both genders (βmen=0.49, βwomen=0.40, p < 0.001), as was access to financial resources (βmen=0.27, βwomen=0.20, p < 0.005), and social power (βmen=0.09, βwomen=0.07, p < 0.05). Differently, equality in participation in full-time work was associated with shorter HLE in both genders (βmen=-0.33, βwomen=-0.30, p < 0.001) and a higher HLE gender gap (β = 0.05, p < 0.05). The HLE gender gap was smaller in contexts with higher equality in access to financial resources (β=-0.06, p < 0.001) and education (β=-0.04, p < 0.01).

**Conclusions:**

These results point to a country-level relation between gender equality and HLE. Beyond the social issue, gender inequalities in education, income and social power seem to play a role in the health of women and men through their aging course.

**Key messages:**

• Higher gender equality in education, income and social power are associated with longer healthy life expectancies in 27 European countries.

• Gender equality benefits not only women’s healthy life expectancies but also men’s, and is reflected in smaller gender gaps.

